# Draft Genome Sequence of Sideroxydans sp. Strain CL21, an Fe(II)-Oxidizing Bacterium

**DOI:** 10.1128/MRA.01444-19

**Published:** 2020-01-09

**Authors:** Rebecca E. Cooper, Carl-Eric Wegner, Sean M. McAllister, Olga Shevchenko, Clara S. Chan, Kirsten Küsel

**Affiliations:** a Aquatic Geomicrobiology, Institute of Biodiversity, Friedrich Schiller University Jena, Jena, Germany; b School of Marine Science and Policy, University of Delaware, Newark, Delaware, USA; c DNA Sequencing and Genotyping Center, University of Delaware, Newark, Delaware, USA; d Delaware Biotechnology Institute, University of Delaware, Newark, Delaware, USA; e Department of Earth Sciences, University of Delaware, Newark, Delaware, USA; f The German Centre for Integrative Biodiversity Research (iDiv) Halle-Jena-Leipzig, Leipzig, Germany; Georgia Institute of Technology

## Abstract

Sideroxydans sp. strain CL21 is an aerobic Fe(II)-oxidizing bacterium isolated from peat sediment from the Fe-rich, moderately acidic Schlöppnerbrunnen fen (northern Bavaria, Germany). Here, we report the draft genome sequence of strain CL21, highlighting genes involved in Fe(II), sulfur, and H_2_ oxidation.

## ANNOUNCEMENT

Sideroxydans sp. strain CL21 is an Fe(II)-oxidizing Gram-negative bacterium that belongs to the *Gallionellaceae* family within the class *Betaproteobacteria*. Isolated from a moderately acidic minerotrophic fen, CL21 can oxidize Fe(II) at pH 4.0 to 6.0 under microaerobic conditions (1). Like its close relative, Sideroxydans lithotrophicus ES-1, it was isolated as a chemolithoautotrophic Fe(II) oxidizer, though strain CL21 growth can be augmented with organics, including lactate. There are relatively few terrestrial Fe(II)-oxidizer isolate genomes, so we sequenced and analyzed the CL21 genome.

*Sideroxydans* sp. CL21 stock cultures were first cultivated at room temperature in the dark in semisolid gradient tubes containing 1% agarose-stabilized modified Wolfe’s minimal medium (MWMM), a defined freshwater medium, as previously described (1, 2), with Fe^0^ as the Fe source and 1 mM Na-lactate. Cultures were transferred to 250-ml serum bottles containing 100 ml MWMM amended with 10 ml liter^−1^ Wolfe’s vitamin solution, 10 ml liter^−1^ trace mineral solution, 10 mM MES (2-[*N*-morpholino]ethanesulfonic acid) buffer (pH 5.5), 1 mM lactate, and an Fe^0^ bottom-layer plug (10 ml MWMM, 3% agarose [PanReac Applichem agarose low EEO {electroendoosmosis} {agarose standard}], 100 mg 10 ml^−1^ Fe^0^). To maintain microaerobic conditions, the headspace was continuously flushed with N_2_:CO_2_:O_2_ at a ratio of 78:20:2 (flow rate, 300 ml min^−1^). Biomass was harvested by centrifugation (10 min, 10,000 × *g*, 4°C), and genomic DNA was extracted using a standard phenol-chloroform-based protocol (3). Whole-genome sequencing was performed on the PacBio RS II platform (Menlo Park, CA) according to the standard manufacturer’s protocol. Briefly, a 10- to 20-kb library was prepared and sequenced on the PacBio RS II sequencer using C4-P6 chemistry on single-molecule real-time (SMRT) cells, with a 180-min collection protocol. Sequence reads were filtered and assembled *de novo* with Hierarchical Genome Assembly Process v4 (HGAP4) using default parameters, except for the seed coverage (25×), seed length cutoff (15,000 bp), and estimated genome size (3.0 Mbp) (4), and annotated with RASTtk (v2.0) using default parameters (5–7). After HGAP4 assembly, the subread count was 129,903, comprising 1,021,527,933 bp. The mean subread length and *N*_50_ value were 5,649 bp and 7,803 bp, respectively.

The draft genome of CL21 was assembled into 1 contig with 263-fold average coverage, a total sequence length of 3.77 Mbp, and a GC content of 54.9%. The sequence quality was assessed with CheckM (v1.0.13) using default parameters (8), which detected 411 of 418 single-copy marker genes only once and 3 single-copy genes twice, equating to 99.37% completeness and 0.79% redundancy. The draft genome contains 3,795 coding sequences (CDS), 52 RNA-coding genes, and 2 16S rRNA genes. Homologs of the Fe(II) oxidation genes *mtoAB* and *cyc2* were identified in *Sideroxydans* sp. CL21 (9). Additionally, genes involved in O_2_ reduction, CO_2_ fixation (RuBisCO), organic C utilization, sulfate respiration, sulfur oxidation, and hydrogen utilization were identified. Taken together, this genome sequence analysis shows that *Sideroxydans* sp. CL21 couples Fe(II) oxidation to assimilation of either inorganic or organic carbon compounds, which are particularly important metabolic processes in organic matter-rich environments like the Schlöppnerbrunnen fen.

### Data availability.

The sequencing reads and assemblies for this whole-genome shotgun project are available in the European Nucleotide Archive (ENA) repository under the BioProject accession number PRJEB33828. The version described in this paper is the first version. The individual genome assembly is available under the accession number LR699166.
